# Ethical Decision-Making and Clinical Ethics Support in Italian Neonatal Intensive Care Units: Results from a National Survey

**DOI:** 10.3390/healthcare14020181

**Published:** 2026-01-11

**Authors:** Clara Todini, Barbara Corsano, Simona Giardina, Simone S. Masilla, Costanza Raimondi, Pietro Refolo, Dario Sacchini, Antonio G. Spagnolo

**Affiliations:** 1 Department of Healthcare Surveillance and Bioethics, Section of Bioethics and Medical Humanities, Università Cattolica del Sacro Cuore, 00168 Rome, Italysalvatoresimone.masilla@unicatt.it (S.S.M.); dario.sacchini@unicatt.it (D.S.);; 2 Fondazione Policlinico Universitario A. Gemelli IRCCS, 00168 Rome, Italy; barbara.corsano@unicatt.it; 3 Research Center for Clinical Bioethics & Medical Humanities, Università Cattolica del Sacro Cuore, 00168 Rome, Italy

**Keywords:** clinical ethics, clinical ethics consultation, neonatal intensive care unit, ethical dilemmas, medico-legal issues

## Abstract

Background/Objectives: Neonatal Intensive Care Units (NICUs) constitute a highly complex clinical environment characterized by patient fragility and frequent ethically sensitive decisions. To date, systematic studies investigating how Italian NICUs address these challenges and what forms of ethics support are effectively available are lacking. The aim of this study is therefore to assess how ethical issues are managed in Italian NICUs, with particular attention to the availability, use, and perceived usefulness of clinical ethics support in everyday practice. Methods: A 25-item questionnaire was developed by adapting an existing tool for investigating clinical ethics activities to the neonatal context. Following expert review by the GIBCE (Gruppo Interdisciplinare di Bioetica Clinica e Consulenza Etica in ambito sanitario), the final instrument covered four areas (general data, experience with ethical dilemmas, tools and procedures, opinions and training needs). A manual web search identified all Italian NICUs and their clinical directors, who were asked to disseminate the survey among staff. Participation was voluntary and anonymous. Data collection was conducted via Google Forms and analyzed through qualitative thematic analysis. Results: A total of 217 questionnaires were collected. The most frequent ethical dilemmas concern quality of life with anticipated multiple or severe disabilities (72.4%) and decisions to withdraw or withhold life-sustaining treatments (64.5%). Major challenges include fear of medico-legal repercussions (57.6%) and communication divergences between physicians and nurses (49.8%). More than half of respondents (52.1%) reported no formal training in clinical ethics, and 68.7% had never developed a Shared Care Plan (Shared Document for healthcare ethics planning) as defined by the Italian Law 219/2017. Conclusions: Findings highlight marked fragmentation in ethical practices across Italian NICUs. On this basis, establishing structured and accessible CEC services could help promote consistency, reinforce shared ethical standards, and support transparent and equitable decision-making in critical neonatal care.

## 1. Introduction

Clinical ethics can be understood as the ethical reasoning that takes shape at the patient’s bedside, where medicine historically found—and continues to find—its most demanding moral questions [1]. It can therefore be described as “a form of applied ethics practiced in the hospital or healthcare settings and concerned with actual clinical choices” [2].

Within this context, clinical ethics services have developed as structures dedicated to the analysis, consultation, and training related to ethical challenges in healthcare practice. Although these services vary across institutional settings, their overarching goal is to support healthcare professionals in decision-making by encouraging interdisciplinary deliberation and ensuring greater transparency in the clinical process [3]. A widely accepted definition of ethics consultation, proposed by the American Society for Bioethics and Humanities (ASBH), describes it as “a service performed by an individual consultant, a team, or a committee to address ethical issues involved in a specific clinical case. Its primary aim is to contribute to patient care, both in process and outcomes, through the identification, analysis, and resolution of ethical problems” [4].

Evidence suggests that integrating clinical ethics into care pathways contributes significantly to improving the quality of care, supporting a person-centered approach, ensuring value-consistency in decisions, and managing moral conflict more appropriately [5].

In the United States and in other Western countries, clinical ethics services are now well established. The Joint Commission International (JCI), a U.S. non-profit organization that accredits healthcare institutions worldwide, requires hospitals to provide a stable reference structure for managing ethical issues, recognizing that inadequate handling of such dilemmas can negatively affect decision-making and clinical outcomes [6]. In Italy, by contrast, despite growing attention to clinical ethics, the system remains highly fragmented with only a limited number of institutions having implemented dedicated Clinical Ethics Consultation (CEC) services [7,8].

The topic of clinical ethics is particularly relevant in neonatology, a highly specialized field characterized by rapid scientific progress and the ongoing evolution of diagnostic and therapeutic options. Clinical situations involving critically ill newborns generate complex ethical questions linked to patient fragility, prognostic uncertainty, and the interplay of professional, family, and societal values.

A major risk in Neonatal Intensive Care (NICU) is the tendency toward either therapeutic neglect or excessive treatment [9]. At the same time, NICUs are also characterized by the “paradoxical” coexistence of palliative and intensive approaches. This coexistence reflects the complexity of neonatal care, where highly technological life-sustaining interventions may need to be integrated with palliative principles aimed at relieving suffering, supporting families, and ensuring the best possible quality of life. Neonatology thus represents a field where clinical decisions often oscillate between curative intentions and the acknowledgement of prognostic uncertainty, thereby intensifying the ethical responsibilities of healthcare professionals [10,11,12,13].

Within this scenario, clinical ethics services represent essential resources to support decision-making and promote justified, proportionate, and participatory clinical practices. In neonatology, participatory decision-making necessarily entails active parental involvement. Although the newborn is the holder of rights, they cannot exercise autonomy [14], nor can clinicians infer personal values from prior expressed preferences. Decisions are therefore made by parents (or legal guardians) and clinicians based on the best interest standard. Initially formulated as a legal principle, the best interest standard has now become a cornerstone of clinical practice, guiding decisions intended to promote a newborn’s overall well-being by integrating medical, prognostic, and value-based considerations [15]. In practical terms, this means avoiding ineffective or disproportionate interventions merely to satisfy parental requests or to comply with defensive medicine. A crucial aspect of applying the best interest standard is the recognition of overtreatment, which should, whenever possible, be prevented through shared planning between the multidisciplinary team and the parents [16]. In this regard, CEC serves as an important resource by providing structured support for ethically sound and participatory decision-making.

Beyond organizational and consultative structures, an often-overlooked dimension of clinical ethics concerns the ethical training and preparedness of healthcare professionals. Empirical evidence suggests that ethical and legal dilemmas related to the limits of life, life-sustaining treatment, and the determination of death are already experienced during education and early professional training, particularly among nurses and midwives [17,18,19,20]. Studies exploring Polish nursing and midwifery students’ perceptions of ethical and legal dilemmas related to brain death have shown significant gaps in the integration of ethical, legal, and clinical knowledge, as well as uncertainty in applying normative frameworks to concrete clinical scenarios [21]. Similarly, research on the conscience clause in brain death care among young nurses and midwives highlights the tension between professional obligations, legal requirements, and personal moral beliefs, demonstrating that ethical challenges are not merely theoretical but deeply embedded in identity formation and professional socialization [22]. Such unresolved ethical tensions have been associated with the early emergence of moral distress, particularly when professionals perceive a mismatch between what they believe ought to be done and what they are institutionally or legally allowed to do [11].

These findings underscore the need for structured and context-sensitive ethics education prior to full clinical responsibility. In the absence of adequate ethical preparation, ethical decision-making risks remaining a normative ideal rather than a sustainable clinical practice. Within NICUs—where prognostic uncertainty, emotional burden, and value conflicts are particularly pronounced—strengthening ethics education and continuous professional training represents a crucial prerequisite for effective ethics support, meaningful parental involvement, and ethically sound decision-making [23].

The aim of this study is to explore how ethical issues arising in neonatal intensive care are addressed within a sample of Italian NICUs. Rather than providing a nationally representative assessment, the study seeks to offer an empirical insight into existing practices, the availability of ethical support tools, and healthcare professionals’ perceptions of their usefulness in this highly complex care setting. By doing so, the study aims to describe current approaches, identify training and educational needs, and highlight areas where ethical support remains insufficient or underdeveloped.

## 2. Materials and Methods

Study design. This study is a descriptive, cross-sectional survey design aimed at exploring clinical ethics activities, ethical dilemmas, and training needs among professionals working in Italian NICUs.

Survey description. To conduct the study, a questionnaire was developed as an adaptation for NICU professionals (including physicians, nurses, psychologists, and other healthcare staff) of an existing instrument designed to investigate clinical ethics activities [7]. The adaptation process involved a preliminary review of the original questionnaire to identify items relevant to the neonatal context, followed by the reformulation of questions to reflect the specific ethical challenges encountered in NICUs. Additional items were created to address issues such as end-of-life decision-making, parental involvement, and therapeutic proportionality. This process resulted in a 25-item multiple-choice questionnaire, complemented by optional open-ended fields for comments.

The draft questionnaire was subsequently discussed and reviewed with members of the GIBCE (Gruppo Interdisciplinare di Bioetica Clinica e Consulenza Etica in ambito sanitario) (https://eticaclinica.wordpress.com/). GIBCE is a research group within the Italian Society of Legal and Insurance Medicine (SIMLA) and is a non-profit association dedicated to promoting the development of clinical bioethics and clinical ethics consultation in Italy.

The questionnaire was conceived as an exploratory and descriptive instrument to collect information on practices and perceptions. It was not designed to measure latent constructs; therefore, no psychometric validation procedures, including internal consistency testing or factor analysis, were performed.

The final version, resulting from this multi-step revision process, is presented in full in Appendix A. The questionnaire comprised four sections: general data, experience with ethical dilemmas, tools and procedures, opinions and training needs.

Data collection procedure. A manual web search identified all Italian NICUs and their respective medical and nursing directors, who were asked to disseminate the survey among their staff. When available, institutional websites provided additional contact information for physicians and nurses.

Participation was voluntary and responses were collected anonymously via Google Forms. All data were handled in full compliance with the GDPR. The survey remained open until 10 November 2025.

A total of 425 emails were sent, with a reminder issued two weeks after the initial contact, on 24 October 2025.

Data analysis. Data analysis was primarily descriptive in nature, in line with the exploratory aims of the study. Responses to closed-ended items were analysed using descriptive statistics, including absolute frequencies and percentages, in order to summarize response distributions across the different sections of the questionnaire.

Although the questionnaire included optional open-ended fields for comments, these responses were limited in number and were not included in the present analysis. Consequently, no formal qualitative analytical framework (e.g., thematic analysis) was applied. The analysis focused on providing an overall descriptive overview of current practices, experiences, and perceived needs related to clinical ethics in NICU settings, without stratification, psychometric testing, factor analysis, or inferential statistical procedures, as these were not consistent with the study objectives.

Ethical approval. The entire study was approved by the Ethics Advisory Board of GIBCE on 27 September 2025.

## 3. Results

A total of 217 questionnaires were completed, yielding a response rate of 51.06%.

Overall, the results provide a descriptive overview of the characteristics of respondents, the frequency and nature of ethical dilemmas encountered in Italian NICUs, the organizational tools available to manage such dilemmas, and professionals’ opinions and training needs.

Below, the findings are presented according to the four sections of the questionnaire.

### 3.1. Section A—General Data

This section describes the main demographic and professional characteristics of the respondents.

Responses were distributed as follows: 46.5% from Northern Italy, 39.2% from Central Italy, and 14.3% from Southern Italy. The majority of respondents were pediatric nurses (58.5%), followed by physicians (36.9%), and psychologists (1.8%). Additional professional roles included nursing coordinators, developmental neuro-psychomotor therapists (TNPEE) and physiotherapists.

Regarding professional experience, 26.3% had worked in NICUs for more than 20 years, 21.2% for over 10 years, 22.1% for 5–10 years, 25.3% for 1–5 years, and 5.1% for less than one year.

### 3.2. Section B—Experience with Ethical Dilemmas

This section highlights the high frequency of ethically challenging situations in NICUs and identifies end-of-life and quality-of-life–related decisions as the most recurrent ethical issues.

The frequency of ethically challenging cases was first examined: 74.2% of respondents reported encountering 1–2 cases per month.

The most frequent ethical issues included quality of life with anticipated severe or multiple disabilities (72.4%), withdrawal or withholding of life-sustaining treatments (64.5%), management of pain and sedation (56.7%), initiation or non-initiation of life support (43.8%) disagreement between parents and multidisciplinary team regarding the newborn’s best interest (32.3%), compassionate extubation (23%), use of experimental or off-label treatments (10.1%), and resource allocation (8.3%).

Respondents also reported several major difficulties in managing ethical dilemmas. The most significant challenges in managing ethical dilemmas were fear of medico-legal repercussions (57.6%), fear of “making the wrong decision” (51.6%), and communication difficulties or divergences between physicians and nurses (49.8%). Communication divergences within the medical team itself were also reported (41%). Additionally, 47.5% reported uncertainty due to the lack of clear legal guidance, and 31.3% cited inadequate institutional support.

When asked which institutional bodies they consulted for ethical dilemmas, respondents indicated: Legal Medicine units (18.9%), Clinical Ethics Committee (17.5%), Bioethics Unit (15.2%), local Ethics Committee (14.7%), and Risk Management units (8.8%). Other responses included Palliative Care teams, external experts, hospital management, and Regional Palliative Care centres. Some respondents indicated that internal medical consultation or the decision of the department head was deemed sufficient.

### 3.3. Section C—Tools and Procedures

This section focuses on the organizational tools, procedures, and resources available for managing ethical dilemmas in NICUs.

Regarding internal procedures for managing ethically challenging cases, 40.1% reported having no dedicated procedure, 14.7% reported having a specific protocol, 11.5% reported having a procedure integrated into a broader protocol, and 33.6% were unaware of any existing procedure.

Professionals typically involved in ethical case discussions included neonatologists (94.9%), psychologists (71.4%), specialist consultants (65.9%), nurses (48.8%), clinical ethics experts (41.5%), legal medicine specialists (22.6%), social workers (21.7%), and chaplains (9.2%).

Parental involvement in decision-making was foreseen by 44.7% for treatment planning and by 44.2% for accepting proposed treatments; in 8.3%, parental involvement was not foreseen.

Time required to reach a joint decision was generally more than one week (44.7%), more than seven days (25.8%), or approximately two days (20.3%). A minority reported that particularly complex cases required several months.

Following case discussion, a written document was produced in 61.8% of cases, whereas 25.8% reported producing no documentation. When documentation was produced, it was filed in the medical record in 65.8% of cases.

Among participants, 29% reported working in a facility with a structured CEC service, while 41.9% were unaware of whether such a service existed. Where a CEC service was available, 53% reported no electronic system for submitting consultation requests, 44.8% did not know such a system existed, and 4.6% reported the use of paper forms only. 50.7% of respondents declared that only physicians were permitted to request an ethics consultation, while 44.8% did not know how to access the service.

Other aspects were also investigated. A total of 77.4% reported having dedicated spaces for meeting with parents. Regarding end-of-life situations, 48.8% reported lacking dedicated spaces for families to spend the final moments with their child, while 35.5% reported having such areas. Some NICUs, described as open-space structures, lacked privacy, while others used temporary isolation rooms when available.

### 3.4. Section D—Opinions and Training Needs

This section summarizes professionals’ views on optimal models for managing ethical dilemmas and their perceived training needs.

When asked how complex cases should ideally be managed, the majority of respondents (69.1%) favored a bedside model supported by a clinical bioethicist or CEC service. Another 24.4% preferred referring cases to an Ethics Committee, while only 2.8% supported bedside assessment without any involvement from a bioethicist.

Nearly all respondents (98.6%) believed that every institution should have a dedicated service for addressing ethical issues.

Regarding training, 52.1% reported that their facility did not offer any form of clinical ethics education.

A total of 68.7% had never participated in Shared Care Planning (Pianificazione Condivisa delle Cure) as defined by Italian Law 219/2017 [24], which requires multidisciplinary collaboration and thorough communication with the patient (or legal representatives) about treatment goals, therapeutic proportionality, possible clinical trajectories, and realistic expectations.

With respect to parental involvement in decision-making, 68.2% did not feel that parental preferences were given excessive weight, and 75.6% reported never having felt that they imposed a decision on parents.

Finally, moral distress was described as occurring “sometimes” (48.8%), “often” (24.4%), and “always” (3.2%).

## 4. Discussion

By examining how Italian NICUs address ethical issues, this survey provides a detailed and up-to-date picture of ethical clinical practices, highlighting both strengths and areas requiring structural intervention. However, because responses predominantly came from Northern and Central Italy—regions with a higher concentration of NICUs—the specific challenges of Southern areas may be under-represented and potentially underestimated [25].

The sample consisted mainly of nurses and physicians, which is consistent with the typical staffing composition of NICUs [26]. A substantial proportion of respondents (over 45%) had more than 10 years of experience, which strengthens the robustness of the findings by grounding them in the perspectives of professionals with long-standing clinical practice and extensive familiarity with ethically complex situations.

Consistent with findings from European and North American NICUs [27,28], three-quarters of participants reported encountering one to two ethical dilemmas per month. This suggests that ethically complex situations are not occasional exceptions but a structural and recurrent component of neonatal intensive care, raising the question of whether current institutional frameworks are adequately equipped to provide timely and systematic ethical support.

The most common ethical issues—quality of life with anticipated severe/multiple disabilities, decisions regarding life-sustaining treatment, and management of pain and sedation—align with literature on neonatal ethics [29]. The reported frequency of disagreements concerning the newborn’s best interest further reflects the ongoing challenges associated with shared decision-making, despite international recommendations [30]. This finding raises important questions about how best to support professionals and families in ethically complex situations, and highlights the need for clearer guidance, structured deliberation, and adequate ethical support within NICUs.

The main barriers reported—including fear of medico-legal consequences, uncertainty, and communication difficulties—reveal a fragile organizational and cultural landscape. The perceived lack of clear legal guidance (47.5%) and limited availability of shared internal procedures may reflect challenges in the practical implementation of Law 219/2017, which requires informed consent, shared care planning, and clinical decisions that carefully balance expected benefits, potential burdens, and the newborn’s best interest through structured communication and documented deliberation with parents or legal representative. Similar challenges have been reported in previous studies [31].

The heterogeneous recourse to Legal Medicine Units, Ethics Committees, and Bioethics Units underscores the absence of standardized national systems for ethical support, with potential implications for the timeliness and coherence of decision-making. This variability raises the question of whether dedicated neonatal ethics pathways or NICU-specific guidelines should be developed at the national level to ensure more consistent and equitable access to ethical support services. In this regard, Anderson et al. [28] emphasize that the presence of clearly identifiable ethics consultation pathways contributes to greater consistency and organizational learning across cases.

Although parental involvement is widely recognized as a cornerstone of neonatal care, the finding that in 8.3% of cases it is not explicitly foreseen raises significant ethical and organizational concerns. This gap appears particularly problematic in light of existing legislative frameworks and international recommendations that strongly promote family-centered care, shared decision-making, and respect for parental roles in clinical deliberations [32]. These findings invite further reflection on the barriers to including parents as partners in decisions and call for additional research to understand whether institutional protocols, professional attitudes, or contextual pressures may hinder effective family participation.

Decision-making often required more than one week, suggesting both the clinical complexity of cases and possible organizational shortcomings. Documentation practices were inconsistent, and only 29% reported the presence of a structured CEC service. Furthermore, the limited availability of digital tools for submitting ethics consultation requests points to a system still developing when compared with international standards [33]. Notably, existing electronic systems typically allow only physicians to request consultations, preventing autonomous access by other professionals. Such structural barriers have been shown to delay ethics consultations and to amplify moral and emotional distress among healthcare professionals [34].

The lack of dedicated spaces for end-of-life situations in nearly half of NICUs represents a remarkable unmet need affecting both parental experience and the quality of relational and emotional support during a highly sensitive time. This finding prompts reflection on how environmental conditions and organizational priorities may shape the experience of dying and bereavement, and whether specific architectural and institutional policies should be implemented to ensure dignified and compassionate end-of-life care.

Healthcare professionals expressed an overwhelming preference for bedside ethics consultation, involving a clinical bioethicist, underscoring the importance of clinical ethics as a practice that takes shape at the patient’s bedside, where ethical reasoning is closely intertwined with real-time clinical decision-making and the specificities of individual cases. This preference mirrors findings reported by Anderson et al. [28], who underline how proximity to the clinical context enhances the perceived effectiveness and legitimacy of ethics consultation services. Overall, this preference aligns with contemporary recommendations that promote integrated, patient-centred clinical ethics services, capable of supporting professionals directly within the care setting rather than through distant or purely advisory mechanisms [35].

The nearly unanimous call for dedicated ethics services (98.6%) and the reported lack of training opportunities reveal a substantial gap between perceived needs and institutional offerings. Similar discrepancies between the demand for ethics support and its effective availability have been documented in other European and North American healthcare systems, where clinical ethics services and ethics education—although more established in some contexts—remain unevenly implemented, variably accessible, and often dependent on local organizational commitment rather than embedded as standard components of care [36,37]. Taken together, these findings suggest that the gap observed in Italian NICUs reflects a broader, transnational challenge in translating the recognized importance of clinical ethics into stable institutional structures and systematic training pathways. The limited implementation of Shared Care Planning, despite its legal grounding, further underscores this discrepancy, suggesting that the formal recognition of ethical and legal principles does not automatically translate into routine clinical practice. Rather, effective implementation appears to depend on organizational commitment, clearly defined procedures, and adequate training, highlighting the need to integrate ethics consultation and education as complementary components of clinical care, particularly in high-complexity settings such as NICUs.

Moral distress, experienced at least occasionally by over 75%, reflects the profound emotional and ethical burden inherent to NICU practice, where high prognostic uncertainty and value-laden decisions are a routine component of care. Rather than representing a secondary or incidental phenomenon, emotional responses appear to play a constitutive role in ethical decision-making processes. As shown by Dahò [34], emotions actively shape both the way ethical issues are framed and the outcomes of clinical ethics deliberation. Within this perspective, moral distress can be understood not only as an individual experience but also as a signal of organizational and ethical strain. The literature suggests that structured ethics consultation processes and adequate ethics training may help mitigate moral distress, improve the ethical climate of care settings, and promote more shared, responsible, and well-informed decision-making [38]. Moreover, the systematic integration of ethics consultation into clinical pathways may serve as a protective factor not only for newborns and families but also for the well-being of healthcare professionals, by reducing the risk of burnout and promoting more sustainable models of care in the long term.

## 5. Limits of the Study

This study has some limitations. The sample size limits the generalizability of the findings. Moreover, although the survey involved professionals from different Italian regions, it cannot be considered a fully representative national survey, as participation was voluntary and not based on a probabilistic sampling strategy. The manual selection of email contacts may have resulted in incomplete coverage of eligible professionals. Furthermore, not all individuals contacted responded, raising the possibility of response bias, whereby professionals with greater interest or sensitivity toward ethical issues may have been more likely to participate. The study relied on a self-administered questionnaire, which may have introduced autocompletion bias. Some questions may also have been interpreted inconsistently by those who received them, particularly given the complexity and context-dependent nature of ethical decision-making in NICUs. Additionally, the study adopts a predominantly Eurocentric perspective, which may limit the applicability of the findings to different cultural or regulatory contexts. Finally, the study relied primarily on closed-ended questions, which, while suitable for providing an exploratory overview of practices and perceptions, may have limited the depth of insight into the underlying reasoning, emotional dynamics, and contextual factors influencing ethical decision-making in NICUs.

Future research could therefore benefit from integrating qualitative approaches, such as interviews or focus groups, to more fully capture the experiential and relational dimensions of ethical challenges in neonatal care. The descriptive nature of the analysis also precludes causal inferences or the identification of predictors associated with specific organizational models or ethical practices. Larger multicentre studies could allow for more robust quantitative analyses, including comparisons across regions, institutional characteristics, or professional roles. Finally, the findings highlight the need for further research aimed at evaluating the impact of structured ethics support services, training programs, and policy interventions on clinical practice in NICUs. Longitudinal and mixed-methods studies may be particularly valuable to assess how different models of ethics consultation, education, and organizational support influence decision-making processes, moral distress, and the quality of care for newborns and families.

These limitations do not undermine the value of the findings but highlight the need for further research involving larger samples and more systematic recruitment methods.

## 6. Conclusions

The aim of this study was to assess how Italian NICUs address ethical issues that arise in clinical practice and, to the best of our knowledge, it represents the first attempt to systematically investigate ethical practices and ethics support structures across a broad sample of neonatal intensive care units in Italy.

Overall, the results portray a system in which ethical sensitivity is widespread but often unsupported by structured tools, specific training, and adequate organizational resources. The survey highlights significant heterogeneity and limited standardization in how Italian NICUs manage ethical dilemmas, as reflected in the variability of reported practices, procedures, and access to ethics support services.

The perceived lack of clear legislative frameworks, the limited availability of structured CEC services, and the scarcity of shared procedures were frequently reported by respondents and appear to hinder systematic and cohesive decision-making processes. The absence of dedicated end-of-life spaces further indicates unmet needs in supporting families during highly delicate moments.

The strong consensus on the importance of dedicated ethics services, combined with the limited training currently offered, suggests a substantial gap between needs and institutional responses. These findings point to areas where organizational development and educational initiatives may be warranted, including the definition of shared procedures and the strengthening of ethics-related competencies among healthcare professionals.

Based on the study findings, it can be hypothesized that a broader national investment in clinical ethics culture—within NICUs and potentially across all healthcare settings—may help promote equity among centers, improve the quality of decision-making, and reduce conflicts and medico-legal disputes. Such an investment could include the development of integrated CEC services, the drafting and adoption of clear shared protocols, and the strengthening of interdisciplinary training, with the potential to support both professional well-being and family-centred care, although further research is needed to evaluate the effectiveness of such interventions.

## Data Availability

The data presented in this study are available from the corresponding author upon reasonable request, subject to ethical approval and data protection requirements.

## Abbreviations

The following abbreviations are used in this manuscript:

NICU	Neonatal Intensive Care Unit
CEC	Clinical Ethics Consultation

## Appendix A. 25-Item Questionnaire

**Section A—General Data**

1. **Where is your hospital located?**

- Northern Italy
- Central Italy
- Southern Italy

2. **What is your profession?**

- Physician
- Nurse
- Psychologist
- Other (please specify)

3. **How many years have you been working in the NICU?**

- <1 year
- 1–5 years
- 5–10 years
- 10 years
- 20 years

**Section B—Experience with Ethical Dilemmas**

4. **On average, how often do ethically challenging cases occur in your NICU?**

- 1–2 per month
- 3–4 per month
- 5 per month

5. **On which topics do ethical dilemmas most frequently arise in your NICU?**
  **(select one or more)**

- Initiation/non-initiation of treatments
- Discontinuation of treatments
- Quality of life with anticipated multiple/severe disabilities
- Pain and sedation management
- Compassionate extubation
- Use of experimental or off-label treatments
- Divergence between parents and clinicians regarding the concept of “best interest”
- Resource allocation
- Other (please specify)

6. **Which factors do you consider most critical in managing an ethical dilemma?**
  **(select one or more)**

- Communication/divergence among physicians
- Communication/divergence between physicians and nurses
- Lack of institutional support
- Fear of making the “wrong decision”
- Fear of medico-legal consequences
- Lack of clear legal guidance
- Other (please specify)

**Section C—Tools and Procedures**

7. **When an ethical dilemma arises, which body do you usually refer to?**

- Territorial Ethics Committee
- Local Ethics Committee
- Clinical Ethics Committee
- Bioethics/Clinical Ethics Unit
- Legal Medicine Unit
- Risk Management Unit
- Other (please specify)

8. **Does your institution have an internal procedure for managing ethically challenging clinical cases?**

- Yes, a specific procedure is in place
- Yes, but it is part of a broader procedure
- No
- I don’t know

9. **Which professional figures are usually involved in discussing a complex clinical case?**
  **(select multiple options)**

- Attending physician (neonatologist)
- Consulting physician (nephrologist, cardiologist, neonatal surgeon, etc.)
- Nurse
- Psychologist
- Social worker
- Case Manager
- Clinical Ethics expert
- Chaplain
- Other (please specify)

10. **Are parents involved in the decision-making process?**

- Yes, to consent to proposed treatments
- Yes, to participate in treatment planning
- No
- Other (please specify)

11. **On average, how long does it take to reach a decision?**

- Within the same day
- 1–2 days
- About one week
- More than one week
- Other (please specify)

12. **Is a document drafted following the discussion?**

- Yes
- No
- Other (please specify)

13. **If yes, is this document attached to the medical record?**

- Yes
- No
- I don’t know
- Other (please specify)

14. **Does your institution have a structured Clinical Ethics Consultation service?**

- Yes
- No
- I don’t know
- Other (please specify)

15. **If yes, is there an electronic system through which a Clinical Ethics Consultation can be requested?**

- Yes
- No
- No, requests can only be made using a paper form
- Other (please specify)

16. **Who is allowed to request a Clinical Ethics Consultation?**

- Physician
- Nurse
- Parents/family members
- Other healthcare professionals
- I don’t know

17. **Are there dedicated spaces in your NICU for meeting with parents?**

- Yes, a dedicated room is available
- No, meetings are usually held at the bedside
- Other (please specify)

18. **Does your NICU have a dedicated room for end-of-life situations, where parents can spend the final moments with their child?**

- Yes
- No
- Other (please specify)

**Section D—Opinions and Training Needs**

19. **In your opinion, complex clinical cases should preferably be addressed:**

- Using a bedside approach among clinicians only
- Using a bedside approach with support from a clinical ethicist/Clinical Ethics Consultation service
- With input from an Ethics Committee
- Other (please specify)

20. **Do you believe that every institution should have a dedicated service for managing ethical issues?**

- Yes
- No
- Other (please specify)

21. **Are training courses in Clinical Ethics offered in your hospital?**

- Yes
- No
- I don’t know

22. **Have you ever had direct experience in drafting a Shared Care Plan (according to the art. 5 of Law 219/2017)?**

- Yes
- No
- Other (please specify)

23. **In your view, in situations of ethical dilemma, is excessive weight sometimes given to parental preferences?**

- Yes
- No
- Other (please specify)

24. **Have you ever felt that you imposed a decision on parents?**

- Yes
- No
- Other (please specify)

25. **To what extent do you experience moral distress in your NICU practice (“knowing the right thing to do, but institutional constraints make it nearly impossible to pursue the right course of action”, Jameton 1984 [39])?**

- Never
- Rarely
- Sometimes
- Often
- Always
